# Carpets and Infection

**Published:** 1892-02

**Authors:** 


### CARPETS AND INFECTION.

Attention is called to this important matter, and all thinking people will at once recognize that in the carpet lurks the germ of many a disease. Frequently a carpet is made necessary on account of the flimsy character of the poorly joined floors, and the accumulations of dirt that fill the crevices, together with the warmth that the carpet affords, form an excellent breeding-place and medium for micro-organisms. Then come the broom and dust-brush which disseminate those germs that were deposited with the dust, and the sunbeam shows us how beautifully the housemaid has aided nature in diffusing the ripe, warm, well-dried, and nourished bacteria.

One can shut his eyes and see the vision of the housemaid, with a towel wrapped around her head, enveloped in a cloud of dust, energetically detaching the myriads of germs from their safe hiding-places; great-grandmothers gloried in their polished floors, and their children inaugurated the wet tea-leaf when the carpet came into general use.

The patent sweeper is a great gain from an hygienic point of view, and if the people will tightly tack down their carpets on a bare floor, they should at least sprinkle it when swept with a patent sweeper, abolish the broom, and once a week wipe up the whole floor with a cloth well saturated with a solution of carbolic acid, creolis, Platt's chloride, or some other such material.

Once a year every carpet in the house should go through the steam-cleaner, and the floor should be scoured with boiling water and carbolic soap. An architect should at the present day be thoroughly posted as to the requirements of hygiene; he can be an apostle of health to the same extent as the physician.

Every house should have a well-laid floor with corners rounded, and this should be required by law. In the large cities the plumbing laws and their enforcement are saving lives, but there is no law to attack the lurking germ in the cracks and crevices of the "bonus building." Let such be made.—*Med. News*, December 12, 1891. (*Climatologist.*)

